# Development of a Methodology for Maintenance of Medicinal Plant Genetic Reserve Sites: A Case Study for Lithuania

**DOI:** 10.3390/plants10040658

**Published:** 2021-03-30

**Authors:** Juozas Labokas, Birutė Karpavičienė

**Affiliations:** Laboratory of Economic Botany, Nature Research Centre, LT-08412 Vilnius, Lithuania; birute.karpaviciene@gamtc.lt

**Keywords:** anthropogenic factors, EU habitat type, factor-specific, habitat-specific, in situ conservation, intervention assessment, management intervention, plant population, target species

## Abstract

In the context of climate change, in situ conservation of plant genetic resources is becoming increasingly important as it supports natural (ecological and evolutionary) adaptations of plants to the changing environment. The aim of this study was to synthesize a comprehensive general methodology for the maintenance of genetic reserve sites of medicinal plants based on the analysis of relevant legal documents, literature sources, databases, and authors’ own experience. A methodology was developed for the application of various maintenance measures for target species populations in genetic reserve sites to ensure their long-term sustainability. It uses a systematic approach to the intervention measures by grouping them into habitat-specific and factor-specific ones, and follows the specific principles of biodiversity conservation, such as the concept of ecosystem, priority of in situ conservation, caution and validity of decision-making, and regional approach. An extensive discussion on major intervention measures is provided. The methodology is intended to be used as a tool for the preparation and implementation of management plans of individual medicinal plant genetic reserve sites by the responsible agencies and protected area managers and is directly linked to the implementation of the EU (European Union) Biodiversity Strategy to 2030 at the national level.

## 1. Introduction

Medicinal and aromatic plant species, including crops and their wild relatives, make up 77% of the total species number in the Euro-Mediterranean region [1]. The international trade analysis showed that most medicinal plants are collected in the wild, as are also many of those produced in cultivation [2]. The largest part of medicinal plant species occurs in the Mediterranean region, which is climatically more favorable for species diversity. Nevertheless, the boreal biogeographical region of Europe, to which Lithuania belongs, possesses relatively larger areas of undisturbed and seminatural habitats abundant in wild plant species of medicinal and related uses. In Lithuania, up to 150 species could be considered priority medicinal plant species [3,4]. As an effect/consequence of rapid climate change, species are shifting their distribution ranges and occupying new habitats [5,6]. It has been projected that the most dramatic changes will occur in Northern Europe, where more than 35% of the species composition in 2100 will be new for that region [7]. These shifts will provide one of the largest challenges to natural resource managers and conservation planners [8]. Besides, there are some other factors affecting species richness and changes in biodiversity. Those include changes in land use and loss of habitats due to human activities, and increasing pressure of invasive species. Therefore, there is an urgent need to safeguard the resources of all socio-economically important plant species and actions should be taken before it is too late.

In Lithuania, 34 genetic reserve sites of medicinal plants have been established accommodating more than 120 medicinal plant species. The purpose of establishing genetic reserve sites, as defined by Article 16 of the Law on Protected Areas of the Republic of Lithuania, is to “preserve the resources of genetic material necessary for activities” [9]. The sustainability of plant genetic resources, as biological resources in general, is largely determined by human activities which often are not environmentally friendly. In the context of climate change, in situ conservation of genetic resources is getting increasingly important compared to ex situ storage of seeds or other propagating material, as it supports natural (ecological and evolutionary) adaptations of plants to the changing environment. Therefore, to ensure the sustainability of plant populations in situ, the major challenge is to find out the most appropriate maintenance measures and wisely apply them. In other words, an active, or dynamic conservation should be implemented as opposed to a passive, or static way of conservation when only a protected area is established.

The aim of this study was to synthesize a comprehensive general methodology for the maintenance of genetic reserve sites of medicinal plants. The methodology is intended to be used as a tool for the preparation and implementation of management plans for the individual genetic reserve sites by the responsible agencies and protected area managers and is directly linked to the EU Biodiversity Strategy to 2030 implementation at the national level [10].

## 2. Results

**Figure 1:**
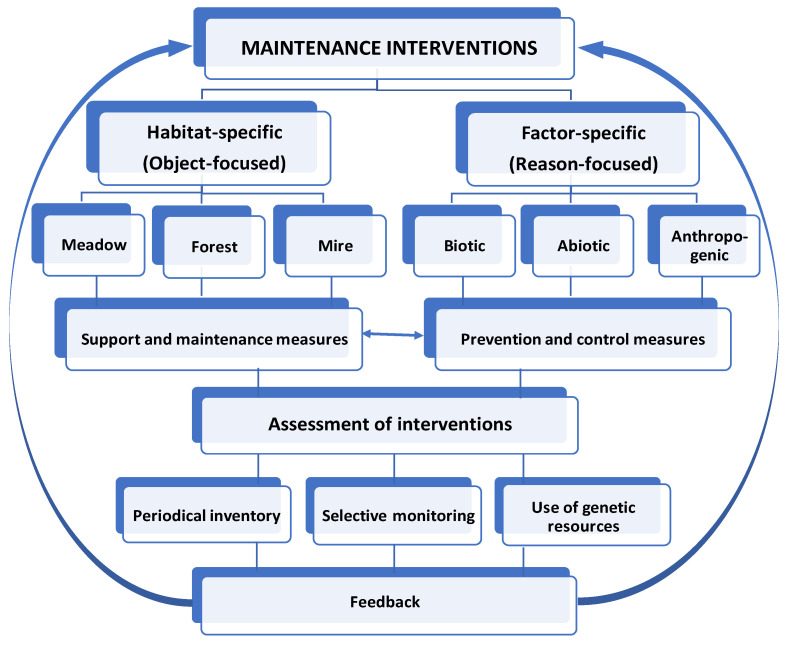
Structure and functioning scheme of maintenance interventions in genetic reserve sites of medicinal plants.

Maintenance of genetic reserve sites presents itself a system of various interventions focused on the long-term sustainability of target species populations, the main object of conservation. It is envisioned to be achieved by maintaining the existing populations and their habitats as close to their natural conditions as possible to make use of ecological and evolutionary adaptations to an ever-changing environment. In general, all maintenance interventions can be grouped into habitat-specific interventions and factor-specific interventions. Habitat-specific interventions are direct object-focused (i.e., population-focused) support and maintenance measures, the degree of applicability of which is predefined by the type of habitat. Here we distinguish three main groups of habitat types: (1) meadow/grassland including heaths and shrub formations, (2) forest, and (3) mire/wetland including bogs, fens, and transition mires. Factor-specific interventions are reason-focused prevention and control measures predefined by the type of factors in effect: biotic, abiotic, or anthropogenic. Naturally, both groups of interventions are interrelated. Finally, an assessment of maintenance interventions is set up with some stipulations on the use and supervision of genetic reserve sites. A schematic illustration of the entire process is presented in Figure 1.

### 2.1. Habitat-Specific Interventions

#### 2.1.1. Meadow Type Habitats (Main Target Species—Herbaceous Plants)

Mowing is carried out at least every two years and not more than once a year. Mowing begins no earlier than July 15 by cutting the grass at a height of 3–5 cm. Cut grass must be removed from the site within two weeks. Mowing machinery should be adequate to the site conditions to avoid soil compression and scraping.

On sites where plant communities have been formed through regular grazing, such as in EU habitat types 6230 * Species-rich *Nardus* grasslands (an asterisk “*” indicates a priority EU habitat type; see Materials and Methods for more details), 6270 * Fennoscandian lowland species-rich dry to mesic grasslands, 5130 *Juniperus communis* formations on heaths or calcareous grasslands, and where no risk of habitat trampling or erosion is posed due to higher soil moisture or surface inclination, grazing may be applied. Grazing intensity, on average during grazing season (from May 1 to October 30), must not exceed one livestock unit (LU) per two hectares (or 0.5 LU/ha). One LU is one animal of cattle or horses or 14 animals of sheep or goats. The lower limit of effective grazing should not be below 0.3 LU/ha. The remainders of ungrazed grass must be mowed by October 15 and removed from the site within two weeks.

Mowing under appropriate conditions (as described above) can be combined with grazing, e.g., mowing in one year and grazing in the other.

When no mowing and no grazing has been applied for more than two or three years, tree and shrub cutting should be considered along with grass mowing or grazing.

Assisted propagation of target species can be applied with the propagation material collected from the same genetic reserve site.

To support target species pollination, beehives can be placed in the genetic reserve sites or nearby during flowering time.

#### 2.1.2. Forest Type Habitats (Target Species of Various Life Forms)

Special, i.e., biodiversity maintenance and other special forest cuttings, as defined by the Regulations on forest cuttings [11], are applied to create better conditions for the development of individuals of target species. These cuttings reduce the number of individuals of non-target species of woody plants, facilitating competition for light, water, and nutrients, as well as thin out too dense target species populations of woody plants. When trees are the main target species, to maintain their genetic diversity and to form self-sustainable stands of different ages and structures, cuttings are carried out to promote fruiting and natural regeneration of trees. The machinery used should not be too heavy to avoid over-compression of forest litter and not damage plants of lower layers. The best time for such interventions is during the winter season when soil is frozen.

In forests on moist or wet substrates, like in habitat types 91D0 * Bog woodland and 9080 * Fennoscandian deciduous swamp woods, the maintenance of the soil moisture regime is of crucial importance. Thus, any drainage attempts should be timely observed and prevented.

Assisted propagation of target species can be carried out with the propagation material collected from the same genetic reserve site.

Genetic reserve sites occurring in EU habitat type 9070 Fennoscandian wooded pastures and alike are maintained by grazing and/or mowing in accordance with the recommendations provided for meadow-type habitats.

For some target tree species (*Tilia cordata* Mill., *Malus sylvestris* Mill., *Prunus* spp., etc.), to support pollination beehives can be placed in the genetic reserve sites or nearby during their flowering time.

#### 2.1.3. Mire Type Habitats (Main Target Species—Herbaceous Plants, Half-Shrubs, and Shrubs)

Soil moisture regime is regularly observed and maintained at the level typical of the mire. Any drainage attempts should be prevented.

Cutting of trees is applicable when a marked increase in the intensity of overgrowth is observed, which is typical in the case of wetland drainage.

Under appropriate conditions (plain surface, not too wet soil) occurring in EU habitat types 7140 Transition mires and quaking bogs and 7230 Alkaline fens, mowing is possible following the recommendations provided for meadow-type habitats.

For some target species (*Menyanthes trifoliata* L., *Rubus chamaemorus* L., *Vaccinium* spp., etc.), to support pollination beehives can be placed in the genetic reserve sites or nearby during their flowering time.

### 2.2. Factor-Specific Interventions

#### 2.2.1. Biotic Factors

Invasive non-native species. To avoid this problem, preventive measures must be applied in the first place: in forest habitats, forest litter must be protected; in meadow habitats, mechanical damages to the sward layer must be avoided, and mire sites should be maintained as natural as they normally are. No cut grass or woody plant residues can be left as well as no fertilizers can be applied in the sites. Plants of invasive species are eradicated, and their populations are controlled by mechanical (hand-pulling, mowing, unearthing, cutting), biological (using herbivores, animal grazing) and, in exceptional cases, chemical (using nationally registered herbicides) means and combinations thereof. Herbicides can only be used against particularly aggressive species, such as Sosnowsky’s hogweed (*Heracleum sosnowskyi* Manden.). In Lithuania, among other control measures, herbicide encapsulations and inoculations are recommended for such woody invasives as *Acer negundo* L., *Amelanchier spicata* (Lam.) K. Koch, *Padus serotina* (Ehrh.) Borkh. and *Robinia pseudoacacia* L. [12].

Problematic native species, also called expansive species. In genetic reserve sites of medicinal plants, wood small-reed (*Calamagrostis epigejos* (L.) Roth) is one of the most problematic species, spreading intensively in unmanaged or improperly managed (mowed grass is not removed) grassland communities. Some less problematic species are common reed (*Phragmites australis* (Cav.) Trin. ex Steud.), cow parsley (*Anthriscus sylvestris* (L.) Hoffm.) and some other nitrophilous plants, which compete strongly with herbaceous and young woody plants. A more frequent selective mowing should be used to control populations of these species, especially when plants are young, with the cut grass removed after each mowing.

When there is a risk of site flooding due to activities of the Eurasian beaver (*Castor fiber* L.), it is controlled that these animals do not dam the streams or drainage ditches in a reserve site or nearby. To protect individual trees from chewing various kinds of fencing and deterring materials can be applied.

Protection of target species from damage caused by deer (*Cervidae*) and other large herbivores is achieved by fencing the sites or by isolating individual, more vulnerable trees, or groups of trees with suitable protective materials (burlap, plastic mesh, cages, etc.) or by using repellents.

In the event of insect pest infestations, nationally registered plant protection products may be used to protect woody plants after a full assessment of their potential effects on the environment in each site. In Lithuania, the list of chemical plant protection products and restrictions on their use are provided by the State Plant Service [13].

Disease prevention is implemented by regular observation of the sites, collecting plant samples when necessary and deciding on measures to be applied.

#### 2.2.2. Abiotic Factors

Flood impact control. As a kind of natural disturbance occurring usually in habitat types 6450 Northern boreal alluvial meadows and 91E0 Alluvial forests with *Alnus glutinosa* and *Fraxinus excelsior* floods are normal habitat-forming factors. A possibility of management intervention might be considered when sites or parts of them remain flooded for an unusually long time or larger than usual areas are flooded.

Drought impact mitigation. This is relevant mostly to grassland habitat types like 6210 Semi-natural dry grasslands and scrubland facies on calcareous substrates, 6230 * Species-rich *Nardus* grasslands., 6270 * Fennoscandian lowland species-rich dry to mesic grasslands and some others. To mitigate drought impact mowing of grass and livestock grazing should be suspended.

Erosion control. The best stabilization of soil is provided by trees, shrubs, and other vegetation. Thus, to prevent soil erosion plant cover should be maintained in erosion-prone sites, like steep slopes, riverbanks, dunes.

#### 2.2.3. Anthropogenic Factors

Fire prevention could be implemented by closing passages to/from the fire-sensitive sites during periods of high fire risk. Public awareness should be raised using mass-media. Warning signs and posters should be employed to inform visitors.

Water regime restoration. This is relevant for the genetic reserve sites which indicate signs of soil desiccation and drop of groundwater level. In such a case site neighborhood should be inspected to identify the reason for undesired drainage impact. Water regime restoration measures are then applied accordingly.

Use of plant resources (harvesting of medicinal plants, berries, nuts, etc.) in genetic reserve sites is permitted in accordance with the Regulations on the use of wild plant resources [14]. Some limitations could be applied to individual sites in relation to the implementation of special conservation and/or monitoring programs.

No feeders and luring spots for ungulates can be established in genetic reserve sites [15].

It must be prohibited to build roads, parking lots, forest warehouses, recreational facilities, hunting installations (towers, stickers, tents, etc.) as well as to change natural ground-water regime (by installing drainage systems) or perform other activities in the areas of genetic reserve sites, which may adversely affect the populations of the target species and their natural recovery.

### 2.3. Assessment of Maintenance Interventions, Use of Genetic Resources and Site Supervision

#### 2.3.1. Inventory and Monitoring

The purpose of a genetic reserve site inventory is to identify and assess changes in the qualitative and quantitative indicators of the populations and habitats of the target species and the factors causing them, to properly plan and take the necessary measures in a timely manner.

The inventory includes the estimation of target species cover-abundance, identification of actual and potential threats, assessment of the effectiveness of intervention measures, collection of seed samples for ex situ conservation and general assessment of site condition according to the methodology developed for this purpose [16].

Periodicity of inventory. In genetic reserve sites where the main target species are herbaceous plants, the inventory is carried out every 4 years. In the case of forest communities, this period may be longer, but not more than 8 years. When actual or potential threats are identified, inventories are performed more frequently.

The inventory data are used to update passport data of the genetic reserve site and, if necessary, the cartographic material, and are stored in the database of a Coordinating Centre for Medicinal Plants as well as in the Central Information System of the National Plant Genetic Resources. Feedback is provided regarding maintenance interventions to the site managers.

Monitoring of genetic reserve sites is carried out on a selective basis by ecogeographical areas and habitat types using methodologies specifically developed for that purpose. Feedback is provided to the site managers.

#### 2.3.2. Use of Genetic Resources and Site Supervision

Collecting of plant genetic resources (seed, vegetative propagating material, seedlings) maintained in genetic reserve sites and use for research, breeding, propagation, and cultivation purposes are permitted in accordance with bona fide principles, without depleting their populations. Users are encouraged to provide feedback to site managers regarding intervention measures needed based on visual site assessment.

Use of state-protected species is permitted only with the authorization of the Environmental Protection Agency as detailed in the Description of the procedure for the use of protected species [17]. Users are encouraged to provide feedback to site managers regarding intervention measures needed based on visual site assessment.

Protection, use, and restoration of genetic reserve sites are supervised by the Lithuanian State Forest Service of the Ministry of Environment, which is responsible for the management of the Central Information System of National Plant Genetic Resources. The Lithuanian State Forest Service organizes and coordinates the periodic inventories of plant genetic resources in cooperation with the Nature Research Centre and other research institutes and universities.

To facilitate practical implementation, the interventions are estimated by their applicability from very common (5 points) to very rarely used (1 point) or not applicable (0) in genetic reserve site management by habitat type and supporting references are provided (Table 1).

## 3. Discussion

### 3.1. Habitat-Specific Interventions

#### 3.1.1. Mowing and Grazing

Lithuania belongs to the Boreal biogeographic region where the final stage of vegetation succession is forest [43]. Therefore, mowing and grazing are major habitat-forming factors of grasslands in this region and both types of maintenance should mainly be used in meadow habitats, and could also be applied in forest and even in mire habitats. The recommended management practices need to be ecologically similar to the historical land use that has formed the habitats [19]. However, with the establishment of modern large farms and fading out small traditional ones, grazing and mowing of small meadows or natural pastures has been significantly abandoned (Table 2).

A condition in favor of livestock grazing application is compensations paid to the farmers participating in the activity “Extensive grassland management by grazing livestock” of the Lithuanian Rural Development Program’s 2014–2020 measure “Agri-Environment Protection and Climate” [18]. In any case, grazing is quite challenging because exceeding the limits of allowed grazing intensity threatens natural species composition and facilitates the appearance of undesired species, typical of nutrient-rich soils. On the other hand, too low grazing intensity (<0.3 livestock units per hectare, as approved by the Ministry of Agriculture [18]) will render livestock grazing ineffective. However, the impact of grazing depends on the animals used: different animals prefer different plant species and it is recommended to use a combination of different grazing animals in grassland management [43]. Škornik et al. reported that in the North Adriatic region low- or moderate-intensity (4–7 sheep ha^−1^) grazing was the most appropriate treatment since it maintained species richness and typical floristic composition of the pastures [45]. This is in line with our recommendation not to exceed 7 sheep per hectare. Regarding the large farm animals, a recent study from western central Germany concluded that more plant species and more high nature value indicator species were observed on paddocks grazed by horses compared to cattle [46]. The abandonment of traditional farming has also resulted in a significant reduction of haymaking in natural and seminatural grasslands. Therefore, their maintenance by mowing has become a biodiversity conservation activity alone. Furthermore, the earliest recommended mowing term, July 15, does not allow to produce the best quality hay as the grass is overgrown, hard and poorly edible by animals. The above-mentioned type of compensation to the farmers participating in the activity “Management of specific meadows” of the Lithuanian Rural Development Program’s 2014–2020 measure “Agri-Environment Protection and Climate” [18] is reasonable support to implement meadow management by mowing in genetic reserve sites too.

#### 3.1.2. Cutting of Trees and Shrubs

In the boreal biogeographical region, with Lithuania being part of it, the prevailing vegetation type is forests, so trees and shrubs naturally occupy the largest part of the zone. Therefore, cutting of trees and shrubs as an intervention measure is nearly equally expected in all three types of habitats. However, the need for cutting application in meadows and mires indicates that: grassland was abandoned of haymaking or grazing, and wetland was drained. Two types of cuttings are used in genetic reserve sites according to the Regulations on forest cuttings [11]: cuttings for biodiversity maintenance and other special cuttings. The latter type is designed particularly for the maintenance and restoration of forest tree genetic reserves and seed tree stands. Cutting for maintaining biodiversity has the purpose of regulating lighting conditions of berries and medicinal plants growing in forests, improving conditions of protected species and/or species of European Community interest and their habitats, etc.

#### 3.1.3. Assisted Propagation

This kind of intervention is better established in forest tree genetic reserve sites when the self-regeneration of trees is insufficient for normal stand recovery or age structure of stands needs to be diversified to ensure stand sustainability. Assisted propagation may also be applied with rare and threatened herbaceous species. For the recovery of genetic stands, there is a provision in Regulations for natural and complex reserves [15] to use propagation material from the same genetic reserve site. In line with the growing idea of assisted migration as a climate change adaptation strategy for native plant species that are less adaptive or mobile [47], there have been attempts made to reintroduce *Arnica montana* L. in north-eastern Lithuania by using seed from southern Lithuanian populations [48]. A review of the research on *Tilia cordata* Mill. carried out by De Jaegere et al. revealed that in Europe, the genetic diversity of *T. cordata* tends to decrease as latitude increases [49].

#### 3.1.4. Pollination

Polination is an increasingly important element of plant conservation strategies. IPBES (Intergovernmental Science-Policy Platform on Biodiversity and Ecosystem Services) report [50] lists the following threats to pollinators: land-use change, intensive agricultural management and pesticide use, environmental pollution, invasive alien species, diseases, and climate change. It adds that climate change already impacts pollinators both through gradual shifts and extreme weather events [50]. Recently it was reported [22] that all bees forage on a mixture of both flowering plants and tree species. Moreover, honeybees have a detectable preference for foraging on trees, even when sparse [22]. Salonen and Julkunen-Tiitto [23] reported that beekeepers used to transfer their hives from Central Finland to mire areas, mainly to Lapland and Northern Ostrobothnia at the end of May, and that 44% of the pollen grains in the mire honey came from *Vaccinium myrtillus* L., *V. vitis-idaea* L., *V. uliginosum* L. and *V. oxycoccos* L., which grow on mires. Thus, supportive pollination should be considered for all major habitat types by placing beehives in genetic reserves.

There are some other population-focused types of intervention, for example, controlled burning. Suggestions exist on the use of controlled burning to support the regeneration of some species, namely, *Pinus sylvestris* L. after cutting of pinewoods in dry habitats, like 91T0 Central European lichen Scots pine forests [51]. Controlled burning is used in England, particularly on moorland and heathland in conservation management, scrub and reedbed management, and in controlling vegetation to reduce risks posed by wildfires [52]. However, considering the substantial risk of damage to other organism species and absence of burning traditions, we treat this type of intervention in genetic reserve sites still premature.

### 3.2. Factor-Specific Interventions

#### 3.2.1. Invasive Non-Native and Problematic Native Species

Triggered by climate change, invasive species is one of the most challenging issues. Currently, 18 species of vascular plants are considered invasive in Lithuania [53]. Basically, there are two major conditions to prevent the spread of invasive species. First, no biomass (hay, woody cutting residues, etc.) should be left accumulating in sites to avoid soil nitrogen enrichment, and second, no bare soil surface should be exposed. Otherwise, both conditions will provide favorable conditions for the undesired species to establishogram). However, open soil spots are needed for some target species propagation as well. For example, our observations have revealed that seedlings of the forest ecotype of *Arnica montana* can be often found on mineralized belts and forest block lines [54]. This could be justified only by the necessity of the establishment of fire belts as required by the Regulations on forest fire protection [40]. One more point is site location regarding potential pathways of alien species. Particular attention should be paid to the riparian sites because many invasive species are distributed along rivers; sites established along roads and railroads also are at higher risk of invasions [55]. In exceptional cases, controlled burning can be applied for eradication of some problematic species like the common reed (*Phragmites australis* (Cav.) Trin. ex Steud.) as stated in the nature management plan of the lake Žuvintas and its shores [56]. Detailed species-specific recommendations for invasive species control are provided by Gudžinskas and Žalneravičius [12]. Recommendations for both invasive and native problematic species control are provided by Rūsiņa [27].

#### 3.2.2. Prevention of Beavers’ Damage

The most damage caused by Eurasian beavers is due to flooding of sites. Large populations of beavers can also cause significant damage to tree species as they chew and cut down even large trees. There are various tools invented to prevent rivers and streams from beaver dam construction [28]. Multiple methods are proposed for tree protection including various kinds of fencing, sand-painting, and taste-aversive tree protection [29].

#### 3.2.3. Protection from Deer and Other Large Herbivores

A review of the research on ungulate herbivory in forests confirmed that manipulation of such herbivory is often highly influential on tree regeneration and on the abundance, diversity and composition of understory vegetation, although it found few studies of boreal areas and long-term herbivory effects [30]. Data from Central Lithuania reveal that forest undergrowth species *Populus tremula* L., *Quercus robur* L., and *Fraxinus excelsior* L., as well as shrub species *Sorbus aucuparia* L. and *Frangula alnus* Mill. are foraged by cervids mostly, while European bison (*Bison bonasus* L.), reintroduced in Lithuania in early 1970s, prefer soft deciduous tree species (*Populus tremula* L., *Alnus incana* (L.) Moench) [32]. Most recently, it was reported that fences or other physical barriers best control for the effects of deer, adding that facilitation by surrounding vegetation, logging slash, hunting, habitat management through timber harvest, and certain repellents may also be moderately effective [31].

#### 3.2.4. Pest and Disease Control

It should be noted that chemical plant protection products are primarily used in the agricultural sector, but some of them may also be used in forestry. A useful source of information, in this regard, is the EU Pesticides Database [57]. However, the prevention measures should be of the first consideration. In forest sites, for example, it is very important to support the natural regeneration of trees as it is more resilient to environmental stresses and reduces the likelihood of introducing new pests with plants for planting. The Food and Agriculture Organization (FAO) promotes integrated pest management which can be defined as a combination of prevention, observation and suppression measures that can be ecologically and economically efficient, and socially acceptable [58]. There are national regulations on forest sanitary protection to be followed by forest managers, owners, and users [33].

#### 3.2.5. Flood Impact Control

An adequate water regime is one of the most important abiotic factors for ecosystem sustainability. Management interventions related to floods are relevant at the riverine sites mostly. Flood water can make damage when sites remain flooded for an unusually long time. Some proposals exist to solve or to mitigate the problem. One idea is to modify the water regime by controlling the flow in the links connecting the floodplain depressions to the river and to each other [34].

#### 3.2.6. Drought Impact Mitigation

One of the most reasonable solutions to mitigate drought impact is canceling of mowing of grass and livestock grazing. In extreme cases, watering in some areas can be considered. For that purpose, natural small water retention measures which combine drought mitigation, flood protection, and biodiversity conservation, are very relevant [35]. According to this source, the construction of micro reservoirs on ditches and restoration of small ponds have the most meaningful impacts on water resources and biodiversity. However, to avoid or minimize any negative impacts on the environment this type of intervention, particularly the construction of micro reservoirs, should be carefully assessed regarding local environmental conditions before implementation. There were some studies done on fertilizer effects on plant drought tolerance and productivity. For example, Australian researchers reported that compost addition to grasslands could be a beneficial management strategy to improve soil health and increase plant productivity, and most importantly to reduce N loss compared to mineral fertilizers [36]. However, adding compost might have some negative effects by facilitating the introduction of seeds of unwanted species including invasive ones.

#### 3.2.7. Erosion Control

Natural, or non-anthropogenic, soil erosion is not a considerable threat to genetic reserve sites in countries with flat topography, like in the case of Lithuania and the other Baltic States. In the past, wind-induced erosion was a problem on open sandy soils in the coastal region of the Baltic sea until it has been controlled by introducing mountain pine (*Pinus mugo* Turra) in 1800s [59]. A good example of that is a narrow land strip of Curonian Spit in Western Lithuania, overgrown largely with mountain pine, the thickets of which are being gradually reduced to prevent fire spread and restore natural open space landscapes [60]. Another issue is water-induced riverbank erosion which wears away sections of banks of some rivers. These processes are hardly controlled and just should be considered before establishing genetic reserve sites. Seasonal or heavy rains caused floods of rivers can also cause soil erosion. In such a case, the mitigation of soil erosion and sediment entry to the river system was demonstrated by simulating the introduction of conservation management practices, vegetation, and riparian buffer strips [36].

#### 3.2.8. Fire Prevention

Regarding meadow-type habitats burning of uncut grass is forbidden by the environmental requirements [37] as well as by the requirements for good agricultural and environmental conditions of agricultural lands [38], the latter stating that grass should not be burned in pastures or meadows. In forest habitats, fire prevention is subject largely to the regulations on forest fire protection [39]. It states, among other things, that the State Forest Enterprise and Directorates of State Nature Reserves and Biosphere Reserves must install fire belts, water bodies, roads for fire-fighting vehicles to reach them and the existing natural water bodies as well as organize the education of the population, i.e., the general public, on the issues of forest fire safety. Mineralized belts of 2–4 m widths, in which combustible substances have been removed, are used to prevent the spread of potential fires including underground fires in drained peat soils. Although fire can promote population growth of several endangered species by reducing litter or by creating and maintaining open habitats [61], a lack of adequate experience with prescribed fire management does not allow its application in genetic reserve site management.

#### 3.2.9. Water Regime Restoration

For the sites established in or next to wetlands, the maintenance of high groundwater level is of critical importance. With the growing necessity of carbon sequestration, projects on degraded peatland restoration or rewetting are being implemented. The experience obtained could be applied in the maintenance of genetic reserve sites suffering from water drainage by building peat dams on the ditches [41].

#### 3.2.10. Control of Wild Plant Harvesting

Locations of genetic reserve sites may often coincide with the sites rich in harvestable plant resources. Then the potential conflict issues may arise between the conservation of plant genetic resources and the use of their physical resources. Although the Regulations on the use of wild plant resources [14] cover timings, forms and other things related to sustainable use of wild plants, some limitations may be introduced covering the implementation of special conservation and monitoring programs of plant genetic resources or with the purpose of site restoration and resource replenishment after unfavorable years. Wild plant harvesting depends much on the market demand, demographic situation (affected largely by emigration) and yearly meteorological conditions. However, wild berry and medicinal plant collecting continue to present themselves as traditional values and an additional source of income [62]. More than half of the respondents of a survey carried out by the authors [62] stated that they needed more information on how to correctly collect raw plant material without causing harm to nature. In Lithuania, the most popular species among wild berry pickers are bilberries (*Vaccinium myrtillus* L.), cowberries (*V. vitis-idaea* L.), cranberries (*V. oxycoccos* L.), wild raspberries (*Rubus idaeus* L.), and wild strawberries (*Fragaria vesca* L.). The total amount of purchased wild berries in 2012 was 1,637,371 kg, with 91% of bilberries. Meanwhile, medicinal plant raw materials totaled 8294 kg [42]. No official statistics on wild-harvested plant product purchase is available for more recent years. Lithuanian State Forest Service data on exports of wild berries (total amount of fresh cranberries, bilberries, cowberries) over the last decade showed its peak of 1,379,000 kg in 2014 and a plunge to 300,000 kg in 2016 with 945,000 kg in 2018 [63]. This indicates that wild plant resources are, in general, not overharvested.

### 3.3. Assessment of Maintenance Interventions, Use of Genetic Resources and Site Supervision

#### 3.3.1. Inventory and Monitoring

Field inventories include botanical and ecological site inventories. Botanical inventory aims at identifying changes in the target and other species cover and abundance as well as health and general condition of the target species populations. Ecological site inventory aims at detecting changes in habitat conditions, and the factors behind, as well as identifying actual and potential threats. A systematic and explicit list of threats is provided at the Reference Portal for Natura 2000 [64]. The assessment of the effectiveness of applied intervention measures along with general site assessment according to the methodology developed for this purpose [16] will provide feedback for further planning and updating maintenance interventions and taking timely management actions.

As stated by the Law on Wild Vegetation [65], to monitor and assess the condition of wild vegetation and its changes, monitoring of wild vegetation is performed as a component of environmental monitoring. Although the State Environmental Monitoring Program 2018–2023 [66] focuses more on threatened, invasive and other problematic species, it also includes a provision on monitoring the condition of vegetation in relatively natural forest ecosystems. Monitoring of genetic reserve sites should be carried out on a selective basis by ecogeographical areas and habitat types using specifically developed methodologies for that purpose. One methodological source to be consulted is the Manual for Integrated Monitoring developed by Finnish Environment Institute [67]. Monitoring will also provide feedback for maintenance intervention planning and implementation.

#### 3.3.2. Use of Genetic Resources and Site Supervision

As there are no specific regulations on collecting plant genetic resources in genetic reserve sites, from the formal point of view one should follow the Regulations on the Use of Wild Plant Resources [14]. However, generally collecting and use of plant genetic resources are subject to the bona fide principle, one of vital importance to all genetic resource users [68]. The use of state-protected species consists basically of their use for research purposes and usually deals with small amounts of plant material. This use is controlled by the Environmental Protection Agency following the Description of the procedure for the use of protected species [17]. All users should provide feedback to site managers.

In Lithuania, plant genetic resources are under the domain of the Ministry of Environment. Therefore, the protection, use, and restoration of genetic reserve sites is supervised by the Lithuanian State Forest Service of the Ministry of Environment, which is responsible for the management of the Central Information System of National Plant Genetic Resources. The State Forest Service organizes and coordinates periodic inventories of plant genetic resources and cooperates with the Nature Research Centre and other state research institutes and universities.

## 4. Materials and Methods

This methodology was based on analysis of relevant legal documents, literature sources and databases as well as authors’ own experience and historical land use that has formed the habitats in Lithuania. It systematically presents measures for the maintenance and protection of genetic reserve sites, aimed at the long-term sustainability of target species populations of medicinal plants. It complies with the Law on Protected Areas [9], Law on National Plant Genetic Resources [69], and other legal acts of the Republic of Lithuania including Regulations on Seed Sites Attributed to National Plant Genetic Resources [70].

**Figure 2:**
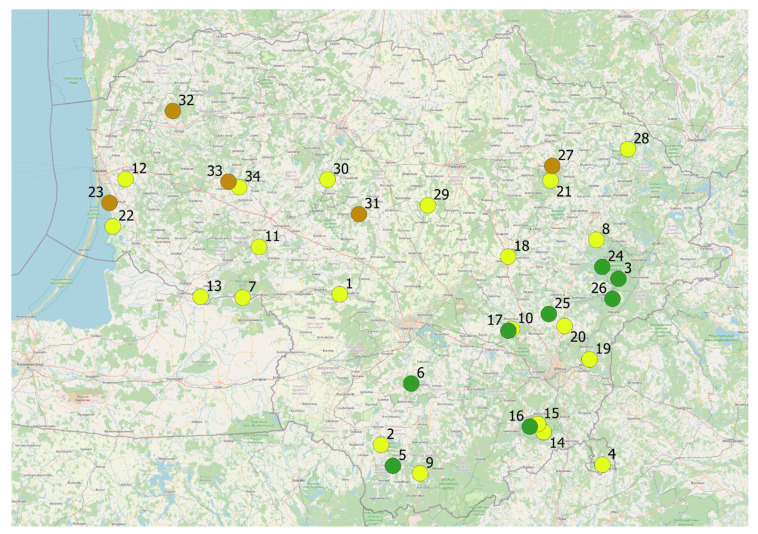
Distribution of 34 medicinal plant genetic reserve sites in Lithuania. Prevailing habitat types: yellow circles ■—meadows; green circles ■—forests; brown circles ■—mires.

A long-term experience on the development of Lithuanian national network of genetic reserve sites for in situ conservation of medicinal and aromatic plant genetic resources acquired by the authors [3], as well as knowledge obtained through the development of guidelines for their evaluation [16], served as the impetus for the current study. In total, 34 genetic reserve sites of medicinal plants have been established since 2006, 31 of which are approved by the Ministry of Environment so far [71] and are subject to maintenance interventions. The sites accommodate more than 120 priority species of medicinal plants (Table S1). Their sizes vary from 0.4 to 38.0 ha with an average of 7.2 ha. Site distribution by prevailing habitat type is as follows: meadows—21 sites or 61.8%; forests—8 sites or 23.5%; and mires—5 sites or 14.7% (Figure 2). The term “medicinal plant”is being used in its wider sense, including such overlapping uses as spices, food, dietary supplements, and cosmetics [72].

Specific principles of biodiversity conservation, such as the concept of ecosystem, priority of in situ conservation, caution and validity of decision-making, regional approach [73], were followed.

For general provisions on management interventions in genetic reserves, Maxted et al. were consulted [74]. Specific provisions were adopted from the existing guidelines for habitat management [75], regulations and procedures approved for the maintenance of meadows [18], forest cuttings [11], invasive species control [24,26,27], use of wild plant resources [14], etc., as well as relevant research papers and data portals. Information on natural habitats of European Community importance in Lithuania was adopted from Rašomavičius [76]. English names of habitat types correspond to the Interpretation Manual of European Union Habitats, version EUR 28, where an asterisk (*) indicates a priority habitat [77]. As the source of information on names of authors of species names Species 2000 & ITIS Catalogue of Life, 2019 Annual Checklist was used [78].

The results are presented in numbered subsections following the structural scheme of maintenance interventions presented in Figure 1, summarized along with applicability estimation of interventions in Table 1 and then discussed correspondingly in a wider context in Discussion.

## 5. Conclusions

The systematic approach to genetic reserve site maintenance presented above contributes to the planning and application of intervention measures in genetic reserve sites by the better perception of the entire process centered on conservation object (populations of target species) and factors (biotic, abiotic, and anthropogenic) affecting it. Nevertheless, the recommended measures should be treated in a flexible, pragmatic manner, considering different target species requirements, local conditions, and potential impacts on the environment by preparing individual site-specific management plans. The individual plans, in turn, should be revised and updated according to the results of periodic inventories and monitoring as well as feedback from the genetic resource users and protected area managers.

It is notorious that anthropogenic factors, including indirect human impacts, are the most critical in ecosystem conservation. The exploitation of natural resources, particularly when seeking the most profitable ones, is among that kind of anthropogenic factor. Meanwhile, wild medicinal plants usually coexist with more profitable natural resources, such as wood and peat, and human interests often focus on the exploitation of the latter, while ignoring medicinal plant conservation efforts. Thus, success of the conservation is predefined much by human attitude towards natural resources. In any case, sites that are out of interest to the other natural resource users or are protected by nature itself (river islands, slopes of hills and valleys, etc.) can be successfully maintained with much less effort. This way a significant part of the resources can be lost. Therefore, it is of critical importance to cooperate with the European ecological network NATURA 2000. It provides several advantages, including better protection of sites and compensation mechanisms to landowners. A standardized habitat classification system and explicit listing of threats, pressures and activities related to their maintenance are additional benefits contributing much to the development of protected site management plans including medicinal plants’ genetic reserve sites.

## Figures and Tables

**Table 1:** Applicability estimation of major genetic reserve site management interventions by habitat type. Estimation scale: 5—very common; 4—common; 3—moderately common; 2—rare; 1—very rare; 0—not applicable.

Type of Intervention	Habitat	Estimation	Type of Supporting Data Source	Reference
Mowing/cutting of grassland	Meadow	5	Regulations on the implementation of the measure “Agri-environment and Climate”; Research paper	[18,19]
	Forest	2	Regulations on the use of wild plant resources	[14]
	Mire	2	Management handbook	[20]
Livestock grazing	Meadow	3	Regulations on the implementation of the measure “Agri-environment and Climate”; Research paper	[18]
	Forest	2	Regulations on the use of wild plant resources	[14]
	Mire	1	Management handbook	[20]
Cutting of trees and shrubs	Meadow	3	Management recommendations	[21]
	Forest	3	Regulations on forest cuttings	[11]
	Mire	2	Management handbook	[20]
Assisted propagation	Meadow	1		
	Forest	2	Regulations on natural and complex reserves	[15]
	Mire	1		
Pollination support	Meadow	5	Research paper	[22]
	Forest	4	Research papers	[22,23]
	Mire	3	Research paper	[23]
Prevention of invasive species	Meadow	5	Management recommendations General program. Database on invasive species General program. Database on invasive species	[21] [24,25] [24,25]
	Forest	5		
	Mire	5		
Control of invasive species	Meadow	5	General procedures. Species-specific recommendations. Life+ project	[12,26,27]
	Forest	5		
	Mire	4		
Prevention and control of native problematic species	Meadow	4	Management recommendations Life+ project Life+ project	[21] [27] [27]
	Forest	4		
	Mire	2		
Prevention of beavers’ damage	Meadow	2	Practical solutions Practical solutions	[28,29] [28,29]
	Forest	2		
	Mire	0		
Protection from deer and other large herbivores	Meadow	0		
	Forest	4	Research papers. Dissertation	[30,31,32]
	Mire	0		
Insect pest and disease control	Meadow	1		
	Forest	2	State authorization of plant protection products. Regulations on forest sanitary protection	[13,33]
	Mire	0		
Flood impact control	Meadow	1	Research paper	[34]
	Forest	1		
	Mire	0		
Drought impact mitigation	Meadow	3	Guidelines on natural small water retention measures. Research paper	[35,36]
	Forest	0		
	Mire	0		
Erosion control	Meadow	2	Research paper; Management recommendations	[21,37]
	Forest	1		
	Mire	0		
Fire prevention	Meadow	4	Regulations on environment protection. Requirements for good agricultural and environmental conditions.	[38,39]
	Forest	4	Regulations on forest fire protection.	[40]
	Mire	4	Management handbook	[20]
Water regime restoration	Meadow	1		
	Forest	1		
	Mire	2	Project LIFE Peat Restore; Management handbook	[20,41]
Control of wild plant harvesting	Meadow	4	Regulations on the use of wild plant resources	[14]
	Forest	5		
	Mire	4		
Control of other anthropogenic intrusions	Meadow	4	Law on Protected Areas. Regulations on natural and complex reserves. Law on Special Land Use Conditions	[9,15,42]
	Forest	4		
	Mire	3		

**Table 2:** Changes in numbers of farms holding livestock over the period 2007–2016 (Statistics Lithuania [44]).

Farm Type & Size	2007	2016	Difference 2016–2007	
Holding 1–9 heads of cattle	118,142	49,280	−68,862	−58%
Holding > 100 heads of cattle	540	1062	+522	+97%
Holding sheep	4122	9505	+5383	+131%

## Data Availability

The data presented in this study are available in Table S1: Priority species of medicinal plants.docx.

## Supplementary Materials

The following are available online at https://www.mdpi.com/article/10.3390/plants10040658/s1, Table S1: Priority species of medicinal plants.docx.

## References

[1] Kell S.P., Knüpffer H., Jury S.L., Ford-Lloyd B.V., Maxted N., Maxted N., Ford-Lloyd B.V., Kell S.P., Iriondo J., Dulloo E., Turok J. (2008). Crops and wild relatives of the Euro-Mediterranean region: Making and using a conservation catalogue. Crop Wild Relative Conservation and Use.

[2] Mulliken T., Inskipp C. (2006). Medicinal plant cultivation—Scope, scale and diversity: Results from an initial analysis. Proceedings of the 1st IFOAM International Conference on Organic Wild Production, Teslic, Bosnia and Herzegovina.

[3] Labokas J., Karpavičienė B. (2018). Creation of a network of seed sites for *in-situ* conservation of medicinal and aromatic plant genetic resources in Lithuania. Botanica.

[4] Nature Research Centre Priority Species of Medicinal and Aromatic Plants in Lithuania (the List). http://www.gamtostyrimai.lt/lt/users/viewGroup/id.4/pageId.42.

[5] Scheffers B.R., Pecl G. (2019). Persecuting, protecting or ignoring biodiversity under climate change. Nat. Clim. Chang..

[6] Wallingford P.D., Morelli T.L., Allen J.M., Beaury E.M., Blumenthal D.M., Bradley B.A., Dukes J.S., Early R., Fusco E.J., Goldberg D.E. (2020). Adjusting the lens of invasion biology to focus on the impacts of climate-driven range shifts. Nat. Clim. Chang..

[7] Alkemade R., Bakkenes M., Eickhout B. (2011). Towards a general relationship between climate change and biodiversity: An example for plant species in Europe. Reg. Environ. Chang..

[8] Lawler J.J. (2009). Climate change adaptation strategies for resource management and conservation planning. Ann. N. Y. Acad. Sci..

[9] Lietuvos Respublikos Saugomų Teritorijų Įstatymas, 1993 m. lapkričio 9 d. Nr. I-301 (Suvestinė Redakcija nuo 2020-01-01 iki 2021-04-30) [Republic of Lithuania Law on Protected Areas, 9 November 1993 No. I-301 (Consolidated version from 01/01/2020 to 30/04/2021)]. https://www.e-tar.lt/portal/lt/legalAct/TAR.FF1083B528B7/asr.

[10] European Commission EU Biodiversity Strategy to 2030. https://ec.europa.eu/info/strategy/priorities-2019-2024/european-green-deal/actions-being-taken-eu/eu-biodiversity-strategy-2030_en.

[11] Miško Kirtimų Taisyklės, Patvirtintos Lietuvos Respublikos Aplinkos Ministro 2010 m. Sausio 27 d. įsakymu Nr. D1-79 (Suvestinė Redakcija nuo 2020-09-02) [Regulations on Forest Cuttings, Approved by the Minister of Environment of the Republic of Lithuania, Order No. D1-79 of 27 January 2010 (Consolidated Version from 02/09/2020)]. https://www.e-tar.lt/portal/lt/legalAct/TAR.4A966C7D30EB/asr.

[12] Gudžinskas Z., Žalneravičius E., Vaitonis G. (2017). Invasive plants. Invasive Species in Lithuania.

[13] The State Plant Service under the Ministry of Agriculture Plant Protection Products Authorisation Division. http://www.vatzum.lt/en/activity/fields-of-activity/plant-protection-products-authorisation/.

[14] Laukinės Augalijos Išteklių Naudojimo Tvarka, Patvirtinta Lietuvos Respublikos Aplinkos Ministro 2000 m. Balandžio 27 d. Įsakymu Nr. 173 (Suvestinė Redakcija nuo 2016-07-30) [Regulations on the Use of Wild Plant Resources, Approved by the Minister of Environment of the Republic of Lithuania, Order No. 173 of 27 April 2000 (Consolidated Version from 30/07/2016)]. https://www.e-tar.lt/portal/lt/legalAct/TAR.08F61BE1F810/asr.

[15] Gamtinių ir Kompleksinių Draustinių Nuostatai, Patvirtinti Lietuvos Respublikos Vyriausybės 2008 m. Balandžio 2 d. Nutarimu Nr. 318 (Suvestinė Redakcija nuo 2020-02-04) [Regulations on Natural and Complex Reserves, Approved by the Government of the Republic of Lithuania, Resolution No. 318 of 2 April 2008 (Consolidated Version from 04/02/2020)]. https://www.e-tar.lt/portal/lt/legalAct/TAR.111D79A65EC5/asr.

[16] Labokas J., Karpavičienė B. (2019). Guidelines for evaluation of seed (genetic) sites of medicinal and aromatic plants in Lithuania. Botanica.

[17] Saugomų Rūšių Naudojimo Tvarkos Aprašas, Patvirtintas Lietuvos Respublikos Aplinkos Ministro 2010 m. liepos 15 d. Įsakymu Nr. D1-622 (Suvestinė Redakcija nuo 2020-03-07) [Description of the Procedure for the Use of Protected Species, Approved by the Minister of Environment of the Republic of Lithuania, Order No. D1-622 of 15 July 2010 (Consolidated Version from 07/03/2020)]. https://www.e-tar.lt/portal/lt/legalAct/TAR.47ED1C2412E4/asr.

[18] Lietuvos Kaimo Plėtros 2014–2020 Metų Programos Priemonės “Agrarinė Aplinkosauga ir Klimatas” Įgyvendinimo Taisyklės, Patvirtintos Lietuvos Respublikos Žemės ūkio Ministro 2015 m. Balandžio 3 d. Įsakymu Nr. 3D-254 [Regulations on Implementation of the Measure “Agri-Environment and Climate” of the Lithuanian Rural Development Program 2014–2020, Approved by the Minister of Agriculture of the Republic of Lithuania, Order No. 3D-254 of 3 April 2015 (Consolidated Version from 16/06/2020)]. https://www.e-tar.lt/portal/lt/legalAct/63420fd0d9fb11e4bddbf1b55e924c57/asr.

[19] Dahlström A., Iuga A.-M., Lennartson T. (2013). Managing biodiversity rich hay meadows in the EU: A comparison of Swedish and Romanian grasslands. Environ. Conserv..

[20] McBride A., Diack I., Droy N., Hamill B., Jones P., Schutten J., Skinner A., Street M. (2011). The Fen Management Handbook.

[21] Calaciura B., Spinelli O. Management of Natura 2000 Habitats. 6210 Semi-Natural Dry Grasslands and Scrubland Facies on Calcareous Substrates (Festuco-Brometalia) (*Important Orchid Sites). European Commission Technical Report 2008, 12/24. https://ec.europa.eu/environment/nature/natura2000/management/habitats/pdf/6210_Seminatural_dry_grasslands.pdf.

[22] Donkersley P. (2019). Trees for bees. Agric. Ecosyst. Environ..

[23] Salonen A., Julkunen-Tiitto R. (2012). Characterisation of two unique unifloral honeys from the boreal coniferous zone: Lingonberry and mire honeys. Agric. Food Sci..

[24] Introdukcijos, Reintrodukcijos ir Perkėlimo Programa, Patvirtinta Lietuvos Respublikos Aplinkos Ministro 2002 m. Liepos 1 d. Įsakymu Nr. 352 (Suvestinė Redakcija nuo 2018-07-01) [Introduction, Reintroduction, and Transfer Program, Approved by the Minister of Environment of the Republic of Lithuania, Order No. 352 of 1 July 2002 (Consolidated version from 01/07/2018)]. https://www.e-tar.lt/portal/lt/legalAct/TAR.4F0F24D6E2BF/sfKIEKzteU.

[25] NOBANIS. http://www.NOBANIS.org.

[26] Invazinių Rūšių Kontrolės ir Naikinimo Tvarkos Aprašas, Patvirtintas Lietuvos Respublikos Aplinkos Ministro 2002 m. Liepos 1 d. Įsakymu Nr. 352 (Suvestinė Redakcija nuo 2018-07-01) [Procedures for Control and Eradication of Invasive Species, Approved by the Minister of Environment of the Republic of Lithuania, Order No. 352 of 1 July 2002 (Consolidated Version from 01/07/2018)]. https://www.e-tar.lt/portal/lt/legalAct/TAR.4F0F24D6E2BF/sfKIEKzteU.

[27] Rūsiņa S. (2017). Annex 3. Control of expansive and invasive plant species. In Nature Conservation Agency of Latvia. Project LIFE11 NAT/LV/000371 NAT-PROGRAMME “National Conservation and Management Programme for Natura 2000 Sites in Latvia” Publication Protected habitat management guidelines for Latvia. Volume 3—Semi-Natural Grasslands. https://www.daba.gov.lv/upload/File/Publikacijas_b_vadlinijas/Hab_Manage_Guidelines_2017_3_Grasslands_annex_03.pdf.

[28] Beavers: Wetlands & Wildlife. Manage Flooding: Various Flow Control Devices Help Manage Flooding. https://www.beaversww.org/manage-flooding/.

[29] Beaver Institute Tree Protection. https://www.beaverinstitute.org/management/tree-protection/.

[30] Bernes C., Macura B., Jonsson B.G., Junninen K., Müller J., Sandström J., Lõhmus A., Macdonald E. (2018). Manipulating ungulate herbivory in temperate and boreal forests: Effects on vegetation and invertebrates. A systematic review. Environ. Evid..

[31] Redick C.H., Jacobs D.F. (2020). Mitigation of deer herbivory in temperate hardwood forest regeneration: A meta-analysis of research literature. Forests.

[32] Kibiša A. (2019). Stumbrų, Bizonų ir Elninių Poveikis Ekosistemoms [Effects of Wisents, Bisons and Cervids on Ecosystems]. Ph.D. Thesis.

[33] Miško Sanitarinės Apsaugos Taisyklės, Patvirtintos Lietuvos Respublikos Aplinkos Ministro 2007 m. Balandžio 11 d. Įsakymu Nr. D1-204 (Suvestinė Redakcija nuo 2020-10-17) [Regulations on Forest Sanitary Protection, Approved by the Minister of Environment of the Republic of Lithuania, Order No. D1-204 of 11 April 2007 (Consolidated Version from 17/10/2020)]. https://www.e-tar.lt/portal/lt/legalAct/TAR.BA9557DAF396/asr.

[34] Zsuffa I., Bogardi J.J. (1995). Floodplain restoration by means of water regime control. Phys. Chem. Earth.

[35] (2015). Global Water Partnership Central and Eastern Europe. Natural Small Water Retention Measures Combining Drought Mitigation, Flood Protection and Biodiversity Conservation. Guidelines, Global Water Partnership Central and Eastern Europe. https://www.gwp.org/globalassets/global/gwp-cee_files/idmp-cee/idmp-nswrm-final-pdf-small.pdf.

[36] Ullah M.R., Corneo P.E., Dijkstra F.A. (2020). Inter-seasonal nitrogen loss with drought depends on fertilizer management in a seminatural Australian grassland. Ecosystems.

[37] Volk M., Möller M., Wurbs D.A. (2010). Pragmatic approach for soil erosion risk assessment within policy hierarchies. Land Use Policy.

[38] Aplinkos Apsaugos Reikalavimai Lauko Sąlygomis Deginant Augalus ar jų Dalis, Patvirtinti Lietuvos Respublikos Aplinkos Ministro 1999 m. Rugsėjo 1 d. Įsakymu Nr. 269 (Suvestinė Redakcija nuo 2016-08-01) [Environmental Requirements for the Outdoor Combustion of Plants or Parts Thereof, Approved by the Minister of Environment of the Republic of Lithuania, Order No. 269 of 1 September 1999 (Consolidated Version from 01/08/2016)]. https://e-seimas.lrs.lt/portal/legalAct/lt/TAD/TAIS.85877/asr.

[39] Žemės Ūkio Naudmenų Geros Agrarinės ir Aplinkosaugos Būklės Reikalavimų, Taikomų nuo 2015 Metų, Aprašas, Patvirtintas Lietuvos Respublikos Žemės Ūkio Ministro 2014 m. Gruodžio 5 d. Įsakymu Nr. 3D-932 (Suvestinė Redakcija Nuo 2020-10-10) [Description of the Requirements for Good Agricultural and Environmental Condition of Agricultural Lands Applicable from 2015, Approved by the Minister of Agriculture of the Republic of Lithuania, Order No. 3D-932 of 5 December 2014 (Consolidated Version from 10/10/2020)]. https://www.e-tar.lt/portal/lt/legalAct/bc9ba1407c6311e4abe983995522ea30.

[40] Miškų Priešgaisrinės Apsaugos Taisyklės, Patvirtintos Lietuvos Respublikos Vyriausybės 1995 m. Balandžio 7 d. Nutarimu Nr. 500 (Suvestinė Redakcija nuo 2020-05-01) [Regulations on Forest Fire Protection, Approved by the Government of the Republic of Lithuania, Resolution No. 500 of 7 April 1995 (Consolidated Version from 01/05/2020)]. https://www.e-tar.lt/portal/lt/legalAct/TAR.B17AF00C766B/asr.

[41] LIFE Peat Restore. A Project Funded by LIFE Climate Change Mitigation Programme (2016–2021). News: Water Regime Restoration Works Begun in the Suursoo Restoration Area. https://life-peat-restore.eu/en/water-regime-restoration-works-begun-in-the-suursoo-restoration-area/.

[42] Lietuvos Respublikos Specialiųjų Žemės Naudojimo Sąlygų Įstatymas, 2019 m. Birželio 6 d. Nr. XIII-2166 (Suvestinė Redakcija nuo 2021-01-01) [Republic of Lithuania Law on Special Land Use Conditions, 6 June 2019 No. XIII-2166 (Consolidated Version from 01/01/2021)]. https://www.e-tar.lt/portal/lt/legalAct/420f4dd0927c11e9ae2e9d61b1f977b3/asr.

[43] Kuris M., Remmelgas L., Prižavoite D., Bojārs E., Ruskule A., Fammler H., Navickas K. (2015). Viable Grassland Management—Experience, Challenges and Opportunities Baltic Environmental Forum. https://vivagrass.eu/wp-content/uploads/2016/03/befvivagrasswwwuus.pdf.

[44] Statistics Lithuania. Official Statistics Portal. https://osp.stat.gov.lt/statistiniu-rodikliu-analize#/.

[45] Škornik S., Vidrih M., Kaligarič M. (2010). The effect of grazing pressure on species richness, composition and productivity in North Adriatic Karst pastures. Plant Biosyst..

[46] Schmitz A., Isselstein J. (2020). Effect of grazing system on grassland plant species richness and vegetation characteristics: Comparing horse and cattle grazing. Sustainability.

[47] Williams M.I., Dumroese R.K., Haase D.L., Pinto J.R., Wilkinson K.M. (2013). Growing assisted migration: Synthesis of a climate change adaptation strategy. National Proceedings: Forest and Conservation Nursery Associations.

[48] (2014). Lietuvos Gamtos Fondas. Kalninę Arniką Siekiama Grąžinti į Aukštaitiją. https://www.glis.lt/?pid=1&news_id=391.

[49] De Jaegere T., Hein S., Claessens H. (2016). A review of the characteristics of small-leaved lime (*Tilia cordata* Mill.) and their implications for silviculture in a changing climate. Forests.

[50] Potts S.G., Imperatriz-Fonseca V.L., Ngo H.T., IPBES (2016). The Assessment Report of the Intergovernmental Science-Policy Platform on Biodiversity and Ecosystem Services on Pollinators, Pollination and Food Production.

[51] NATURA 2000 Prioritetinių Veiksmų programa (PVP), Skirta Įgyvendinti Lietuvoje Pagal Tarybos Direktyvos 92/43/EEB Dėl Natūralių Buveinių ir Laukinės Faunos bei Floros Apsaugos (Buveinių Direktyvos) 8 Straipsnį [NATURA 2000 Priority Action Program for Implementation in Lithuania in Accordance with Article 8 of Council Directive 92/43/EEC on the CONSERVATION of Natural Habitats and of Wild Fauna and Flora (Habitats Directive)]. http://am.lrv.lt/uploads/am/documents/files/saugom_teritorijos_kra%C5%A1tov/natura_2000/PAF-2019-03-27.pdf.

[52] The Heather and Grass Burning Code 2007 Version. https://gfmc.online/programmes/natcon/UK-DEFRA--Heather-Grass-Burning-Code-2007.pdf.

[53] Invazinių Lietuvoje Rūšių Sąrašas, Patvirtintas Lietuvos Respublikos Aplinkos Ministro 2004 m. Rugpjūčio 16 d. Įsakymu Nr. D1-433 (Suvestinė Redakcija nuo 2016-12-24) [List of Invasive Species in Lithuania, Approved by the Minister of Environment of the Republic of Lithuania, Order No. D1-433 of 27 August 2004 (Consolidated Version from 24/12/2016)]. https://www.e-tar.lt/portal/lt/legalAct/TAR.7B6390A69C91/asr.

[54] Radušienė J., Labokas J., Maxted N., Ford-Lloyd B., Kell S., Iriondo J., Dulloo E., Turok J. (2008). Population performance of *Arnica montana* L. in different habitats. Crop Wild Relative Conservation and Use.

[55] Ascensão F., Capinha C., Borda-de-Água L., Barrientos R., Beja P., Pereira H. (2017). Aliens on the move: Transportation networks and non-native species. Railway Ecology.

[56] Žuvinto Ežero ir jo Pakrantės Gamtotvarkos Planas, Patvirtintas Lietuvos Respublikos Aplinkos Ministro 2016 m. Spalio 24 d. Įsakymu Nr. D1-704 [Nature Management Plan of the Lake Žuvintas and Its Shores, Approved by the Minister of Environment of the Republic of Lithuania, Order No. D1-704 of 24 October 2016]. https://www.e-tar.lt/portal/lt/legalAct/322ad9509c0411e69ad4c8713b612d0f.

[57] European Commission EU Pesticides Database. https://ec.europa.eu/food/plant/pesticides/eu-pesticides-db_en.

[58] FAO (2011). Good Practices for Forest Health Protection. In FAO Forestry Paper 164 Guide to Implementation of Phytosanitary Standards in Forestry. Food and Agriculture Organization of the United Nations Rome. http://www.fao.org/docrep/013/i2080e/i2080e03.pdf.

[59] Jørgensen H. NOBANIS—Invasive Alien Species Fact Sheet—*Pinus mugo*—From: Online Database of the European Network on Invasive Alien Species—NOBANIS. https://www.nobanis.org/globalassets/speciesinfo/p/pinus-mugo/pinus_mugo.pdf.

[60] Jurgutavičiūtė K. Kirtimai Kuršių Nerijoje—Gamtos ir Kraštovaizdžio Labui. http://nerija.am.lt/VI/article.php?article_id=1044.

[61] Deák B., Valkó O., Török P., Végvári Z., Hartel T., Schmotzer A., Kapocsi I., Tóthmérész B. (2014). Grassland fires in Hungary‒Experiences of nature conservationists on the effects of fire on biodiversity. Appl. Ecol. Environ. Res..

[62] Karvelytė A., Motiekaitytė V. (2013). Assessment of Wild Mushrooms, Berries and Medicinal Herbs Picking Activities by Referring to Economic, Social and Environmental Criteria. J. Young Sci..

[63] Lithuanian State Forest Service (2019). Multiple Use of Forests. In Forestry Statistics 2019 (pdf). http://www.amvmt.lt/images/veikla/stat/miskustatistika/2019/07%20Misku%20ukio%20statistika%202019_m.pdf.

[64] European Environment Agency (2000). Threats, Pressures, Activities. In Eionet Central Data Repository. Reference Portal for Natura. http://cdr.eionet.europa.eu/help/natura2000.

[65] Lietuvos Respublikos Laukinės Augalijos Įstatymas, 1999 m. birželio 15 d. Nr. VIII-1226 (Suvestinė Redakcija nuo 2004-02-28 iki 2021-04-30) [Republic of Lithuania Law on Wild Vegetation, 15 June 1999 No. VIII-1226 (Consolidated Version from 28/02/2004 to 30/04/2021)]. https://www.e-tar.lt/portal/lt/legalAct/TAR.B810DCE56C74/asr.

[66] Valstybinė Aplinkos Monitoringo 2018–2023 Metų Programa, Patvirtinta Lietuvos Respublikos Vyriausybės 2018 m. Spalio 5 d. Nutarimu Nr. 996 (Suvestinė Redakcija nuo 2021-01-01) [State Environmental Monitoring Program 2018–2023, Approved by the Government of the Republic of Lithuania, Resolution No. 996 of 5 October 2018 (Consolidated Version from 01/01/2021)]. https://www.e-tar.lt/portal/lt/legalAct/d6fdb4b0c89a11e8bf37fd1541d65f38/asr.

[67] Finnish Environment Institute (1998). Manual for Integrated Monitoring.

[68] Cooper D., Engels J., Frison E. (1994). A Multilateral System for Plant Genetic Resources: Imperatives, Achievements and Challenges. Issues Genet. Resour..

[69] Lietuvos Respublikos Augalų Nacionalinių Genetinių Išteklių Įstatymas, 2001 m. Spalio 9 d. Nr. IX-533 (Suvestinė Redakcija nuo 2019-05-01) [Republic of Lithuania Law on National Plant Genetic Resources, 9 October 2001 No. IX-533 (Consolidated Version from 01/05/2019)]. https://www.e-tar.lt/portal/lt/legalAct/TAR.55D93E8A9C77/asr.

[70] Sėklinių Sklypų, Kurie Priskiriami Augalų Nacionaliniams Genetiniams Ištekliams, Nuostatai, Patvirtinti Lietuvos Respublikos Aplinkos Ministro 2003 m. Gruodžio 10 d. Įsakymu Nr. 631 [Regulations on Seed Sites Attributed to Plant National Genetic Resources, Approved by the Minister of Environment of the Republic of Lithuania, Order No. 631 of 10 December 2003]. https://www.e-tar.lt/portal/lt/legalAct/TAR.D86B5DD7C643.

[71] Augalų Nacionaliniams Genetiniams Ištekliams Priskirtų Vaistinių ir Aromatinių Augalų Sėklinių (Genetinių) Sklypų sąrašas, Patvirtintas Lietuvos Respublikos Aplinkos Ministro 2009 m. Gruodžio 31 d. Įsakymu Nr. D1-861 (Suvestinė Redakcija nuo 2020-06-06) [List of Genetic Reserve Sites of Medicinal and Aromatic Plants Attributed to National Plant Genetic Resources, Approved by the Minister of Environment of the Republic of Lithuania, Order No. D1-861 of 31 December 2009 (Consolidated Version from 06/06/2020)]. https://www.e-tar.lt/portal/lt/legalAct/TAR.AAF6299E727A/asr.

[72] European Commission European Red List. Introduction to Medicinal Plants. https://ec.europa.eu/environment/nature/conservation/species/redlist/med_plants/introduction.htm.

[73] Marozas V. (2008). Sausumos Ekosistemų Įvairovė ir Apsauga: Vadovėlis.

[74] Maxted N., Iriondo J.M., De Hond L., Dulloo E., Lefevre F., Asdal A., Kell S.P., Guarino L., Iriondo J.M., Maxted N., Dulloo M.E. (2008). Genetic reserve management. Conserving Plant. Genetic Diversity in Protected Areas: Population Management of Crop. Wild Relatives.

[75] Rūsiņa S., Auniņš A., Lārmanis V., Spuņģis V. (2017). Annex 2. Optimal, Suboptimal and Inappropriate Management of EU Protected Habitats. In Nature Conservation Agency of Latvia. Project LIFE11 NAT/LV/000371 NAT-PROGRAMME “National Conservation and Management Programme for Natura 2000 sites in Latvia” publication Protected Habitat Management Guidelines for Latvia. Volume 3—Semi-Natural Grasslands. https://www.daba.gov.lv/upload/File/Publikacijas_b_vadlinijas/Hab_Manage_Guidelines_2017_3_Grasslands_annex_02.pdf.

[76] Rašomavičius V. (2012). EB Svarbos Natūralių Buveinių Inventorizavimo Vadovas.

[77] European Commission (2013). Interpretation Manual of European Union Habitats, Version EUR 28. http://ec.europa.eu/environment/nature/legislation/habitatsdirective/docs/Int_Manual_EU28.pdf.

[78] Roskov Y., Ower G., Orrell T., Nicolson D., Bailly N., Kirk P.M., Bourgoin T., DeWalt R.E., Decock W., Nieukerken E. (2019). Species 2000 & ITIS Catalogue of Life, 2019 Annual Checklist.

