# Supplementary material for: Development of a Methodology for Maintenance of Medicinal Plant Genetic Reserve Sites: A Case Study for Lithuania
Source: Plants (Basel). 2021 Mar 30;10(4):658. doi: 10.3390/plants10040658 (PMC8065864; doi:10.3390/plants10040658)
Supplement: Supplementary file 1 [file plants-10-00658-s001.pdf]

plants MDPI

Table S1. Priority species of medicinal plants

Achillea millefolium L.	Geranium robertianum L.	Pulsatilla pratensis (L.) Mill.
Acorus calamus L.	Geum rivale L.	Quercus robur L.
Agrimonia eupatoria L.	Geum urbanum L.	Rhamnus cathartica L.
Alchemilla vulgaris L.	Glechoma hederacea L.	Ribes nigrum L.
Allium angulosum L.	Gratiola officinalis L.	Rosa canina L.
Allium oleraceum L.	Helichrysum arenarium (L.) Moench	Rubus caesius L.
Allium scorodoprasum L.	Hepatica nobilis Mill.	Rubus chamaemorus L.
Allium ursinum L.	Herniaria glabra L.	Rubus idaeus L.
Allium vineale L.	Hippophae rhamnoides L.	Rubus nessensis Hall
Alnus glutinosa (L.) Gaertn.	Humulus lupulus L.	Rubus plicatus Weihe & Nees
Alnus incana (L.) Moench	Hypericum maculatum Crantz	Rumex acetosa L.
Angelica archangelica L.	Hypericum perforatum L.	Rumex crispus L.
Antennaria dioica (L.) Gaertn.	Juniperus communis L.	Rumex thyrsiflorus Fingerh.
Arctostaphylos uva-ursi (L.) Spreng.	Lamium album L.	Salix ×fragilis L.
Arnica montana L.	Ledum palustre L.	Salix purpurea L.
Artemisia absinthium L.	Leonurus cardiaca L.	Sanguisorba officinalis L.
Artemisia vulgaris L.	Linaria vulgaris Mill.	Saponaria officinalis L.
Berberis vulgaris L.	Lithospermum officinale L.	Scrophularia nodosa L.
Betula pendula Roth	Lycopodium clavatum L.	Sedum acre L.
Betula pubescens Ehrh.	Lycopus europaeus L.	Solanum dulcamara L.
Calluna vulgaris (L.) Hull	Lythrum salicaria L.	Solidago virgaurea L.
Carex arenaria L.	Mentha aquatica L.	Sorbus aucuparia L.
Carum carvi L.	Mentha arvensis L.	Stachys officinalis (L.) Trevis.
Centaurium erythraea Rafn	Menyanthes trifoliata L.	Symphytum officinale L.
Chelidonium majus L.	Myrica gale L.	Tanacetum vulgare L.
Cichorium intybus L.	Oenothera biennis L.	Taraxacum campylodes G.E.Haglund
Comarum palustre L.	Origanum vulgare L.	Thymus pulegioides L.
Convallaria majalis L.	Persicaria bistorta (L.) Samp.	Thymus serpyllum L.
Corylus avellana L.	Picea abies (L.) H.Karst.	Tilia cordata Mill.
Crataegus monogyna Jacq.	Pilosella officinarum Vaill.	Trifolium pratense L.
Dryopteris filix-mas (L.) Schott	Pinus sylvestris L.	Urtica dioica L.
Elymus repens (L.) Gould	Plantago lanceolata L.	Vaccinium myrtillus L.
Epilobium angustifolium L.	Plantago major L.	Vaccinium oxycoccos L.
Equisetum arvense L.	Polemonium caeruleum L.	Vaccinium uliginosum L.
Filipendula ulmaria (L.) Maxim.	Populus tremula L.	Vaccinium vitis-idaea L.
Fragaria vesca L.	Potentilla anserina L.	Valeriana officinalis L.
Fragaria viridis Weston	Potentilla erecta (L.) Raeusch.	Verbascum thapsus L.
Frangula alnus Mill.	Primula veris L.	Veronica officinalis L.
Fraxinus excelsior L.	Prunella vulgaris L.	Viburnum opulus L.
Galium odoratum (L.) Scop.	Prunus padus L.	Vincetoxicum hirundinaria Medik.
Gentiana cruciata L.	Prunus spinosa L.	Viola tricolor L.
